# Monetary value and cost analysis of a youth voluntary program on road safety in Iran 

**Published:** 2019-08

**Authors:** Hamed Seddighi, Ibrahim Salmani, Hamide Seddighi

**Affiliations:** ^*a*^Student Research Committee, University of Social welfare and Rehabilitation Sciences, Tehran, Iran.; ^*b*^Department of Health in Emergency and Disaster, Shahid Sadoughi University of Medical Sciences, Yazd, Iran.; ^*c*^Allame Tabatabaei University, Tehran, Iran.

## Abstract

**Background:**

Volunteering has great economic and social benefits, but it is neglected due to the voluntary ‎nature of the work. The purpose of this study was to assess the monetary aspect of voluntary ‎activities in the plan of Iranian National Safety and Health for New Year Holidays passengers ‎and analyze cost analysis of the implementation of this plan by youth volunteers. ‎

**Methods:**

In this descriptive-analytical study, 1574 volunteers outlined in this plan were analyzed from ‎Wage Replacement and Replacement Cost approach for monetary evaluation of voluntary ‎activities. In addition, the cost benefit analysis of a Red Crescent voluntary plan is calculated by ‎value audit and volunteer investment (VIVA) technique. ‎

**Results:**

In the mentioned road safety plan, the sum of the economic value of volunteering work in the ‎Nowruz passenger s safety and health plan is multiplied by the number of working hours of ‎youth volunteers per day, the number of young people and the average wage was earned at an ‎hour, which was obtained as $ 69885.6. Also Viva rate is 10.6$ means every dollar Red ‎Crescent spent will cost more than 10$ if it wasn’t voluntary. ‎

**Conclusions:**

According to the findings, it seems that the voluntary plan for safety and health of New Year ‎holidays passengers had been economically profitable for the Red Crescent population as well ‎as for the government because of its high revenue compared to the its cost.‎

**Keywords:**

Cost Analysis, Road Safety, Monetary Valuation, Volunteering, Youth, Road safety

